# Rdh10a Provides a Conserved Critical Step in the Synthesis of Retinoic Acid during Zebrafish Embryogenesis

**DOI:** 10.1371/journal.pone.0138588

**Published:** 2015-09-22

**Authors:** Enrico D’Aniello, Padmapriyadarshini Ravisankar, Joshua S. Waxman

**Affiliations:** The Heart Institute, Molecular Cardiovascular Biology and Developmental Biology Divisions, Cincinnati Children’s Hospital Medical Center, Cincinnati, OH, United States of America; Laboratoire de Biologie du Développement de Villefranche-sur-Mer, FRANCE

## Abstract

The first step in the conversion of vitamin A into retinoic acid (RA) in embryos requires retinol dehydrogenases (RDHs). Recent studies have demonstrated that RDH10 is a critical core component of the machinery that produces RA in mouse and *Xenopus* embryos. If the conservation of Rdh10 function in the production of RA extends to teleost embryos has not been investigated. Here, we report that zebrafish Rdh10a deficient embryos have defects consistent with loss of RA signaling, including anteriorization of the nervous system and enlarged hearts with increased cardiomyocyte number. While knockdown of Rdh10a alone produces relatively mild RA deficient phenotypes, Rdh10a can sensitize embryos to RA deficiency and enhance phenotypes observed when Aldh1a2 function is perturbed. Moreover, excess Rdh10a enhances embryonic sensitivity to retinol, which has relatively mild teratogenic effects compared to retinal and RA treatment. Performing Rdh10a regulatory expression analysis, we also demonstrate that a conserved teleost *rdh10a* enhancer requires Pax2 sites to drive expression in the eyes of transgenic embryos. Altogether, our results demonstrate that Rdh10a has a conserved requirement in the first step of RA production within vertebrate embryos.

## Introduction

Vitamin A deficiency (VAD) represents a public health problem in underdeveloped countries (reviewed in [1]). While the incidence of excessive vitamin A (hypervitaminosis A) is rare compared to VAD, the use of vitamin A and its synthetic analogues for the treatment of dermatological diseases or cancer can be problematic, particularly if there is inappropriate self-medication with vitamin A supplements [2, 3]. The importance of proper vitamin A levels to embryonic development first became apparent from studies beginning in the 1940s by Warkany and colleagues, who demonstrated that VAD results in pleiotropic embryonic defects in multiple organs, including the eyes and heart [4–9]. Subsequent to the initial studies on VAD embryos, a series of studies investigated the effects of hypervitaminosis A on mammalian embryos [10–15], finding it is teratogenic and causing a spectrum of conserved defects, including anencephaly, microtia/anotia, micrognathia, cleft palate, cardiac defects, thymic abnormalities, and eye malformations.

**Fig 1:**
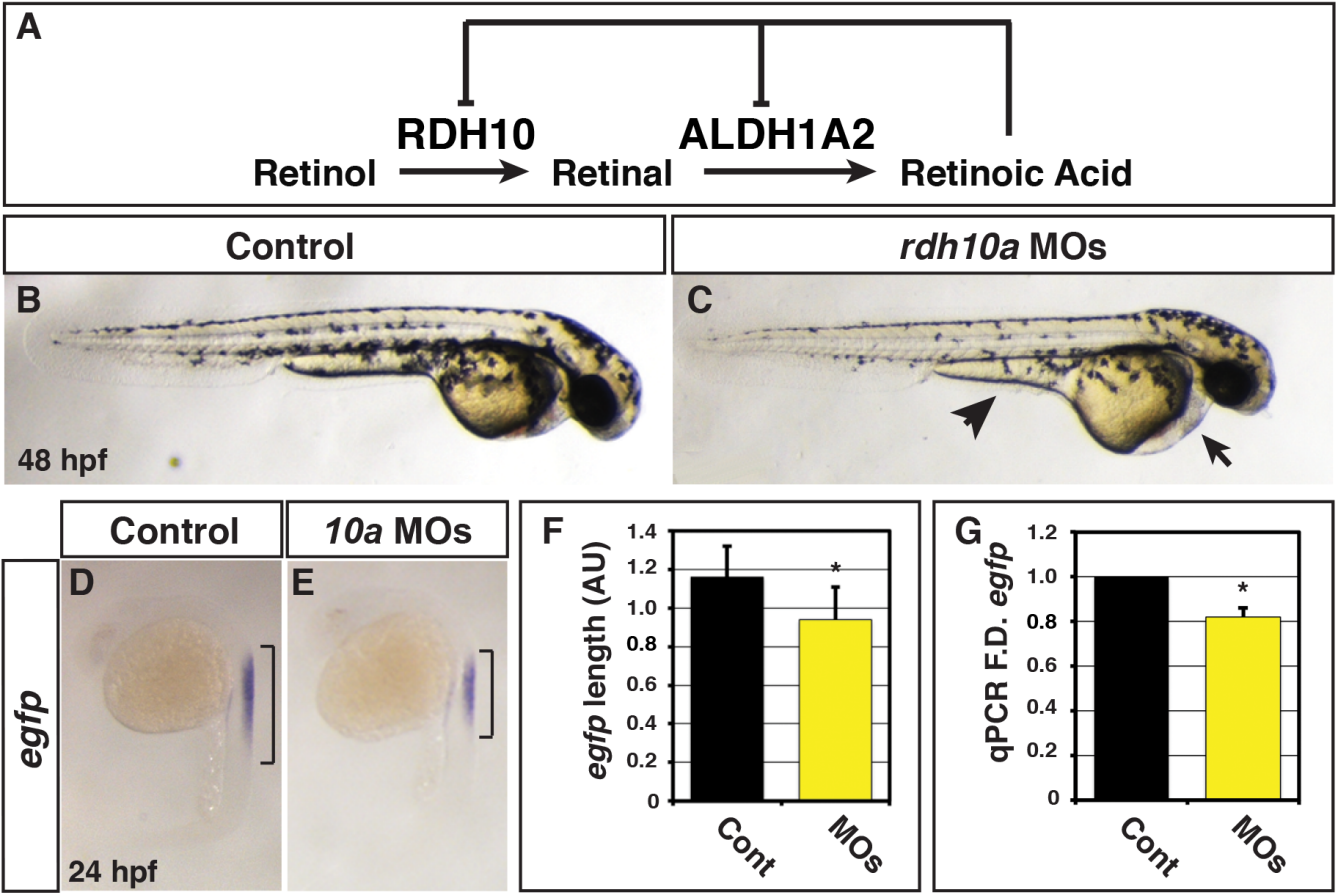
Rdh10a deficient embryos have decreased RA signaling. (A) Schematic of the metabolic pathway that synthesizes RA during development and negative regulation of *Rdh10* and *Aldh1a2* expression from mice and *Xenopus* studies [23, 28, 29]. (B) Control sibling embryo. (C) Embryos injected with *rdh10a* MOs. Arrow indicates pericardial edema. Arrowhead indicates yolk extension. (D-E) ISH for *egfp* expression in *Tg(12XRARE-ef1a*:*EGFP)* ^*sk72*^ control sibling and Rdh10a deficient embryos. Brackets indicate the length of *egfp* expression in the spinal cord. (F) Measurements of *egfp* expression length in the spinal cord using arbitrary units (AU) from *in situ* hybridization (ISH) of *Tg(12XRARE-ef1a*:*EGFP)* ^*sk72*^ control embryos (n = 16) and Rdh10a deficient embryos (n = 15). (G) RT-qPCR for *egfp* expression at 24 hpf in control sibling and Rdh10a deficient embryos. In all graphs, asterisks indicate a statistically significant difference (p<0.05) and bars indicate standard deviation.

Retinoic acid (RA), a potent morphogen during embryogenesis, is the most abundant metabolic product of vitamin A. RA levels must be tightly regulated within tissues during embryogenesis as excessive or insufficient RA signaling can cause pleiotropic congenital abnormalities and fetal death [16–19]. Thus, appropriate embryonic RA signaling relies on the amount of vitamin A available. RA is metabolized from vitamin A/retinol (ROL) through sequential oxidation reactions. In embryos, ROL dehydrogensases (Rdhs), which belong to the short chain dehydrogenase/reductase (SDR) family, convert ROL in retinal (RAL) [20]. Retinaldehyde dehydrogenases (Aldh1as) subsequently oxidize RAL into retinoic acid (Fig 1A) [21] Although it was once thought that Aldh1a enzymes produced RA in specific embryonic locations, while RAL was ubiquitously produced from many different RDH enzymes [22], recent data in mice demonstrated that Rdh10 is required for the majority of RA production during embryogenesis. Rdh10 mutant mice have variable background-dependent defects, with the strongest defects resembling Aldh1a2 mutant embryos [23–26]. Furthermore, Rdh10 has specific locations of embryonic expression in the neural tube, lateral plate mesoderm, and somites that closely parallels expression of *Aldh1a2* [27, 28], the most prevalent embryonic RA producing enzyme. *Xenopus* Rdh10 is expressed in similar tissues as the mouse Rdh10 during early development and is required for the production of embryonic RA, although knockdown of *Xenopus* Rdh10 produces comparably less severe RA deficiency than found in the severe Rdh10 KO mice embryos [23, 28, 29]. Importantly, in mice and *Xenopus*, RA signaling negatively regulates *Rdh10* expression (Fig 1A) [23, 29], suggesting it is part of a critical, conserved embryonic feedback mechanism that regulates embryonic RA levels [30].

Like Rdh10 in mice and *Xenopus*, the zebrafish ortholog Rdh10a has localized expression in the anterior neural tube, lateral plate mesoderm, and somites and is negatively regulated by RA signaling [31–33], suggesting that it may have a conserved role and be the primary RAL-producing enzyme in zebrafish embryos. Here, we investigated the role of Rdh10a in the stepwise synthesis of RA and the consequences of excess ROL during early zebrafish embryogenesis. We found that depletion of Rdh10a results in defects indicative of loss of RA signaling, although the phenotypes observed are less severe than impairment of Aldh1a2 function. Consistent with a role in RA production, we found that Rdh10a genetically interacts with Aldh1a2 and that Rdh10a depletion can overtly enhance the loss of RA signaling phenotypes in Aldh1a2 impaired embryos. Conversely, overexpression of *rdh10a* mRNA can enhance RA signaling and enhance the sensitivity to ROL treatment. However, ROL alone even at high concentrations is significantly less potent than RAL or RA treatment. Lastly, we identified a conserved teleost *rdh10a* enhancer that requires Pax2 binding sites for expression in the eye of transgenic embryos. Altogether, our work establishes that Rdh10a is required for the production of RA in zebrafish embryos, supporting the hypothesis that a conserved core biosynthetic regulatory network controlling RA levels was established early in the vertebrate lineage.

## Results

### Rdh10a deficient zebrafish embryos have decreased RA signaling

Previous studies demonstrated that *rdh10a* is expressed in the ventrolateral mesoderm during gastrulation, the somites during post-gastrulation, and later in the endoderm, hindbrain, optic vesicle and pharyngeal arches of zebrafish embryos (http://zfin.org) [31–33]. *Rdh10b*, its paralog, is expressed mainly in Kuppfer’s vesicle and notochord (http://zfin.org). The location of *rdh10a* expression is more similar to the single *Rdh10* orthologs in mice and *Xenopus* [23, 25, 29, 31]. Furthermore, *rdh10a* expression is significantly more responsive to fluctuations in RA signaling compared to *rdh10b* [31]. Therefore, the locale and sensitivity of *rdh10a* expression to RA levels implies that it may be the predominant Rdh required for embryonic RA production, as is found for the single Rdh10 in mammals and amphibians. To address the function of Rdh10a during zebrafish embryogenesis, we used *rdh10a* translation and splice-blocking morpholinos (MOs) (S1A Fig). While the level of Rdh10a depletion is not known, by 48 hours post-fertilization (hpf), embryos deficient for Rdh10a had pericardial edema and thicker, less tapered yolk extensions reminiscent of Aldh1a2 deficient embryos, but did not show other overt phenotypic defects (Fig 1B and 1C). To determine if RA signaling is affected in Rdh10a deficient embryos, we took advantage of the RA signaling reporter line *Tg(12XRARE-ef1a*:*EGFP)* ^*sk72*^ [34]. We found that Rdh10a deficient embryos have a modest decrease in RA reporter expression at 24 hpf (Fig 1D–1G). To examine the specificity of this phenotype, we injected the *rdh10a* MOs along with *rdh10a* mRNA that should not bind the MOs into the *Tg(12XRARE-ef1a*:*EGFP)* ^*sk72*^ reporter line. Although injection of 200 pg *rdh10a* mRNA alone did not affect *GFP* expression from the reporter, *rdh10a* mRNA was able to improve the expression of the RA signaling reporter in Rdh10a deficient embryos (S2 Fig), supporting the specificity of the phenotype.

**Fig 2:**
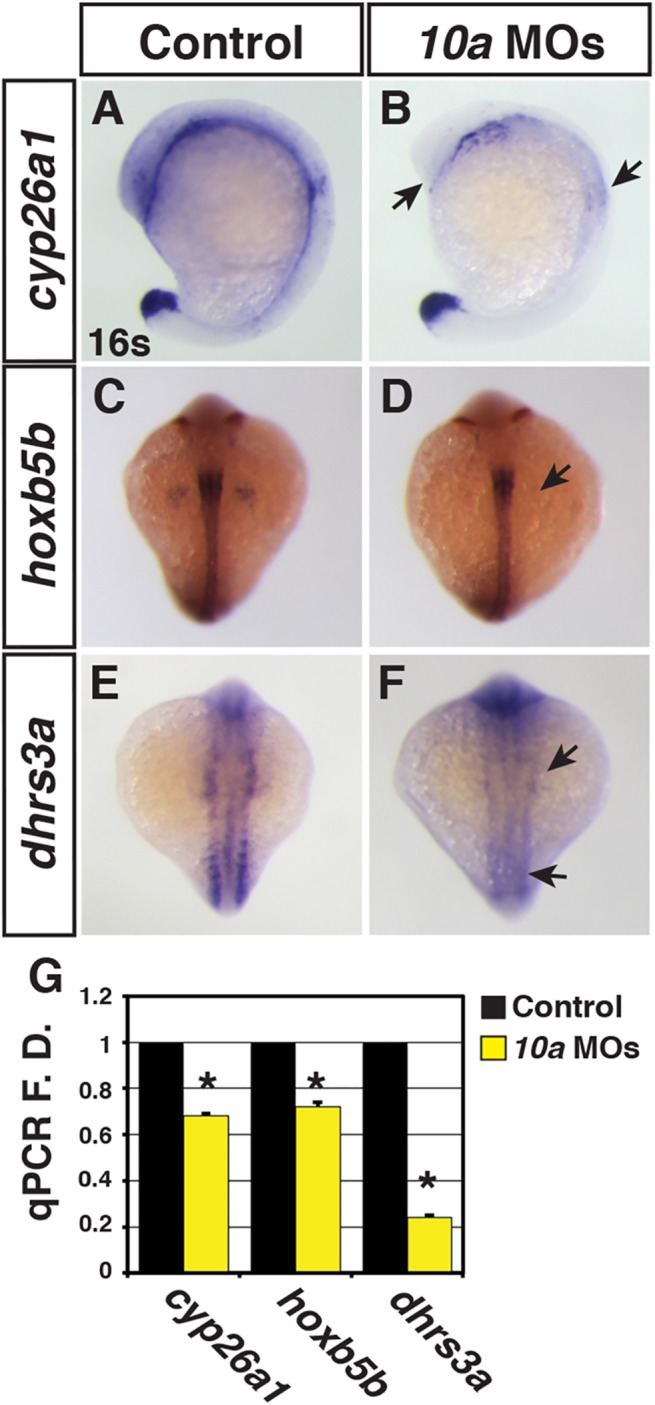
Rdh10a deficient embryos have decreased expression of RA signaling responsive genes. (A-F) ISH for *cyp26a1*, *hoxb5b*, *and dhrs3a* expression in control sibling and Rdh10a deficient embryos. In A and B, views are lateral with dorsal rightward. In C-F, views are dorsal. In all images anterior is up. Arrows in B, D and F indicate decreased expression relative to control sibling embryos. (G) RT-qPCR for RA signaling responsive genes.

**Fig 3:**
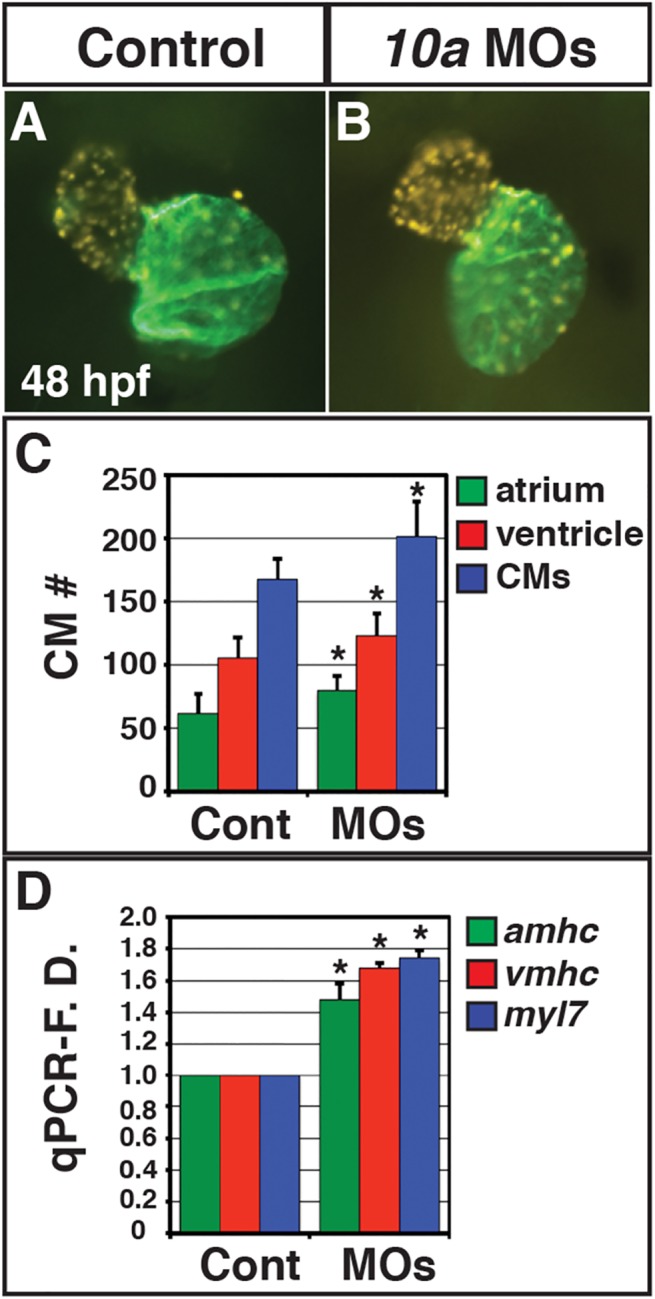
Rdh10a deficient embryos have increased CM number. (A, B) Hearts from control sibling (n = 10) and Rdh10a deficient (n = 10) *Tg(-5*.*1myl7*:*DsRed2-NLS)* ^*f2*^ embryos. Images are frontal views. Red indicates ventricle. Green indicates atrium. (C) Mean CM number at 48 hpf. (D) RT-qPCR for CM differentiation marker gene expression at 48 hpf.

To further understand the impact of Rdh10a depletion on RA signaling, we next examined the expression of positively-regulated RA signaling responsive genes. We found that *cyp26a1*, *hoxb5b* and *dhrs3a* expression were decreased in Rdh10a deficient embryos (Fig 2A–2G), as would be expected from a loss of RA signaling [31, 35–37]. The pericardial edema in Rdh10a deficient embryos (Fig 1B) was indicative that there may be cardiovascular defects in Rdh10a deficient embryos. Since loss of RA signaling can cause an increase in cardiomyocyte (CM) specification [31, 38, 39], we examined the hearts of Rdh10a deficient embryos. Although the morphology of hearts from Rdh10a deficient embryos was not significantly disrupted at 48 hpf (Fig 3A and 3B), counting the number of CMs using *Tg(-5*.*1myl7*:*DsRed-NLS)* ^*f2*^ embryos [38, 40] revealed Rdh10a deficient embryos had increased atrial and ventricular CM number and enhanced expression of CM differentiation marker genes, *myl7 (*pan-cardiac), *vmhc* (ventricular), and *amhc* (atrial) (Fig 3C and 3D), which again are suggestive of increased CM specification found with loss of RA signaling [31, 38, 39]. We conclude that abrogation of Rdh10a decreases RA production and leads to a loss of RA signaling in zebrafish embryos.

### Depletion of Rdh10a sensitizes embryos with impaired Aldh1a2

**Fig 4:**
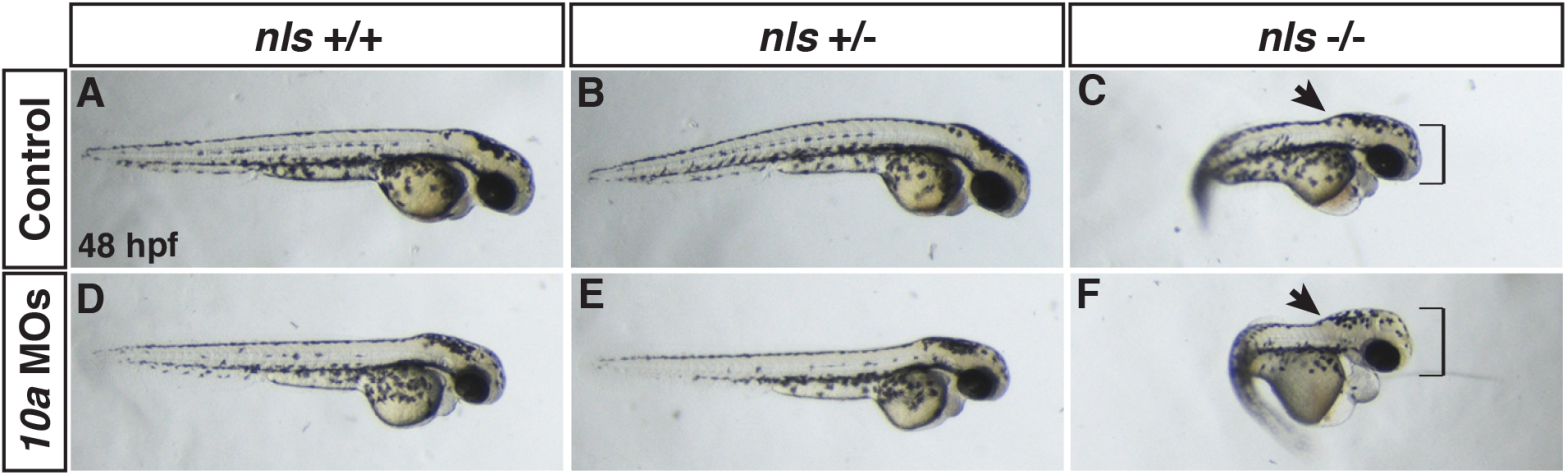
Rdh10a depletion enhances defects in *nls*/*aldh1a2* mutant embryos. (A, D) WT control and Rdh10a deficient embryos. (B,E) Control and Rdh10a deficient embryos heterozygous for the *nls* allele. (C,F) Control and Rdh10a deficient embryos homozygous for the *nls* allele. *Nls* mutant embryos (C) show anteriorization of nervous system (black arrowhead), increases in the size of the head (black bracket), and pericardial edema. Rdh10a deficient; *nls* mutant embryos (F) have an accentuated anteriorization of the nervous system (arrowhead), larger and dysmorphic head (red bracket), and enhanced pericardial edema compared to *nls* embryos. Dorsal is up and anterior is to the right.

Given the relatively mild phenotypes we observed in Rdh10a depleted embryos, we wanted to determine the functional consequences of concurrent depletion with *aldh1a2/neckless* (*nls*) mutant embryos [41], which have more severe RA loss of function phenotypes. While more severe than Rdh10a deficient embryos, the *nls* allele is a missense mutation and may not represent a complete lack of embryonic RA production [41]. To examine the functional interaction between Rdh10a and Aldh1a2, we injected the *rdh10a* MOs in embryos from an in-cross of heterozygous *nls* adult carrier fish. We found that depletion of Rdh10a in *nls* ^*+/+*^ or *nls* ^*+/-*^ backgrounds did not overtly indicate a stronger loss of RA signaling than the MOs alone (Fig 4A, 4B, 4D and 4E). However, when Rdh10a was depleted in *nls* ^*-/-*^ embryos, we observed a visible functional interaction with Rdh10a depleted-*nls* ^*-/-*^ embryos having shorter body axes, enlarged heads, an enhanced boundary between the hindbrain and spinal cord, and more prominent pericardial edema compared with the most severe *nls* ^*-/-*^ embryos (Fig 4C and 4F). To examine this functional interaction in another manner, we also depleted Rdh10a from embryos treated with a suboptimal concentration of DEAB (an Aldh1a inhibitor) [42] that does not cause severe phenotypes. Consistent with our analysis in *nls* mutants, Rdh10a deficient embryos treated with a suboptimal concentration of DEAB were more strongly anteriorized, compared to the suboptimal dose alone (S3 and S4 Figs). Therefore, the functional interaction between Rdh10a and Aldh1a2 supports that Rdh10a is required for the production of embryonic RA in zebrafish embryos.

### Rdh10a is sufficient for the production of RA

**Fig 5:**
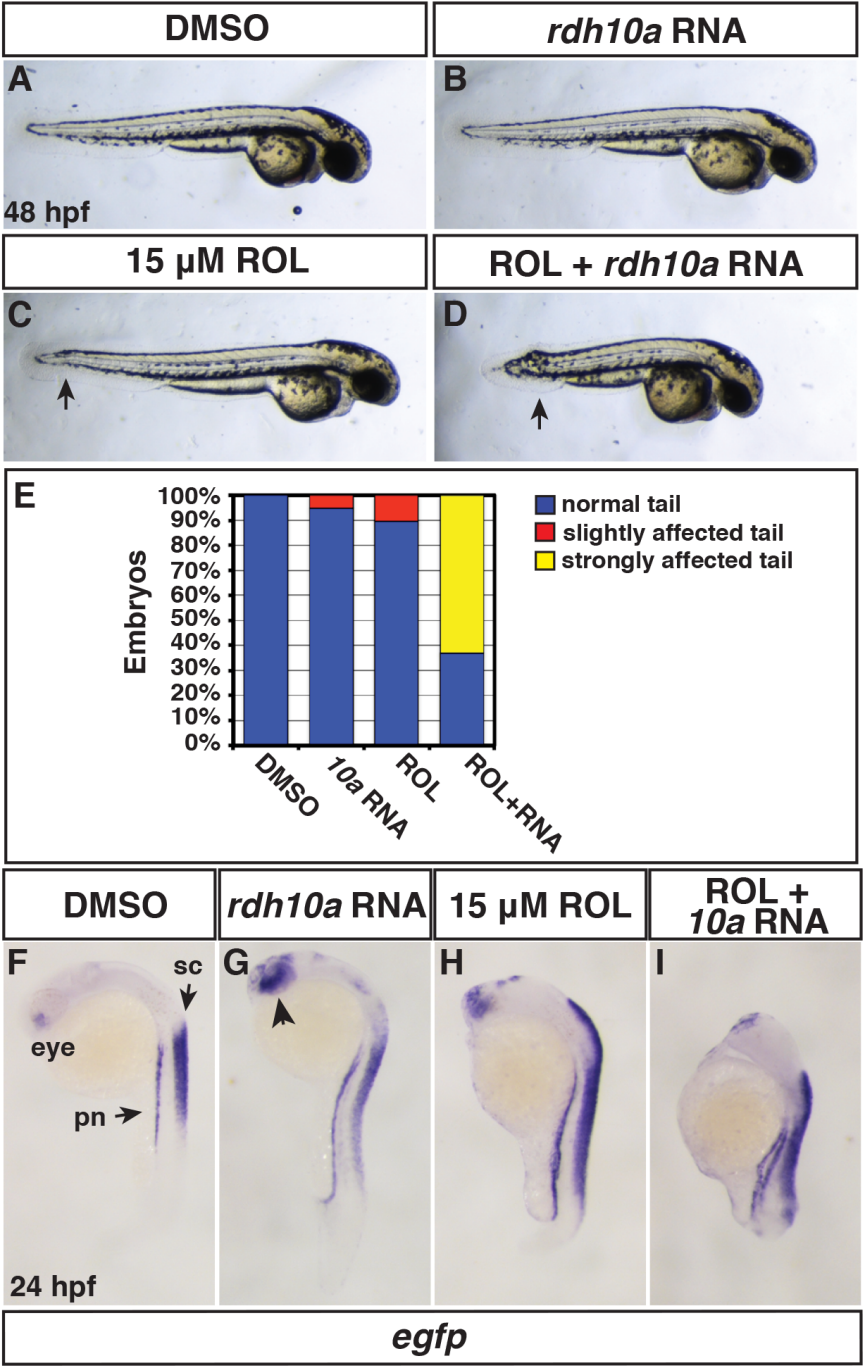
Rdh10a overexpression promotes embryonic RA signaling. (A, F) Control DMSO treated, (B,G) *rdh10a* mRNA (300 pg), (C,H) ROL treated (15 μM), and (D,I) ROL treated, *rdh10a* mRNA injected embryos. (A-D) At 48 hpf, DMSO and *rdh10a* mRNA injected embryos were overtly normal. 15 μM ROL produced overtly normal embryos, except some have a modestly shortened, kinked tail (arrow in C). ROL treated, *rdh10a* mRNA injected embryos were severely truncated. Views are lateral with dorsal up and anterior right. (E) Percentage of truncated embryos at 48 hpf in A-D. For each condition n = 19 embryos. (F-I) ISH for *egfp* expression in *Tg(12XRARE-ef1a*:*EGFP)* ^*sk72*^ embryos. In F, pn indicates pronephros and sc indicates spinal cord. In G, arrowhead indicates anterior neural expression. Views are lateral with dorsal right and anterior up.

We next turned our attention to the consequences of increased *rdh10a* expression. While 200 pg of *rdh10a* mRNA is able to restore Rdh10a deficiency, it did not induce overt phenotypic defects or alter the expression of the RA signaling reporter. However, injection of 300 pg *rdh10a* mRNA, while still not inducing overt phenotypic defects (Fig 5A and 5B), was able to promote ectopic *Tg(12XRARE-ef1a*:*EGFP)* ^*sk72*^ reporter expression in the spinal cord, pronephros, brain, and eyes (Fig 5F and 5G), suggesting that *rdh10a* mRNA overexpression can enhance RA production. The relatively mild phenotype of *rdh10a* mRNA-injected embryos raised the question of whether Rdh10a activity might be restricted by insufficient endogenous ROL concentrations, as RAL, not ROL, is the primary precursor stored in teleost embryos [43, 44]. Therefore, we determined if Rdh10a can enhance the sensitivity to ROL treatment, similar to what has been found in *Xenopus* [29]. We used a dose of ROL (15 μM) that we found had minimal overt effect when treatment was initiated at the sphere stage, but is able to promote expression of the RA signaling transgenic reporter (Fig 5C and 5H). In contrast to injection of *rdh10a* mRNA or ROL treatment alone that did not cause significant overt defects, ROL treatment of *rdh10a* mRNA injected embryos caused severely truncated embryos with tail defects (Fig 5D, 5E and 5I), which is reminiscent of RA treatment and enhanced RA in the tailbud [45, 46]. Interestingly, the tails were more sensitive to ROL-treated *rdh10a* mRNA injection than the anterior of the embryos (Fig 5D), which is very sensitive to RA treatment and usually affected with RA treatment [37, 45, 47]. Therefore, these results suggest that ROL is at least partially limiting in the embryos and that Rdh10a is sufficient to promote increased RA signaling when there is excess ROL in zebrafish embryos.

### Comparison of the teratogenicity of excess retinoids

**Fig 6:**
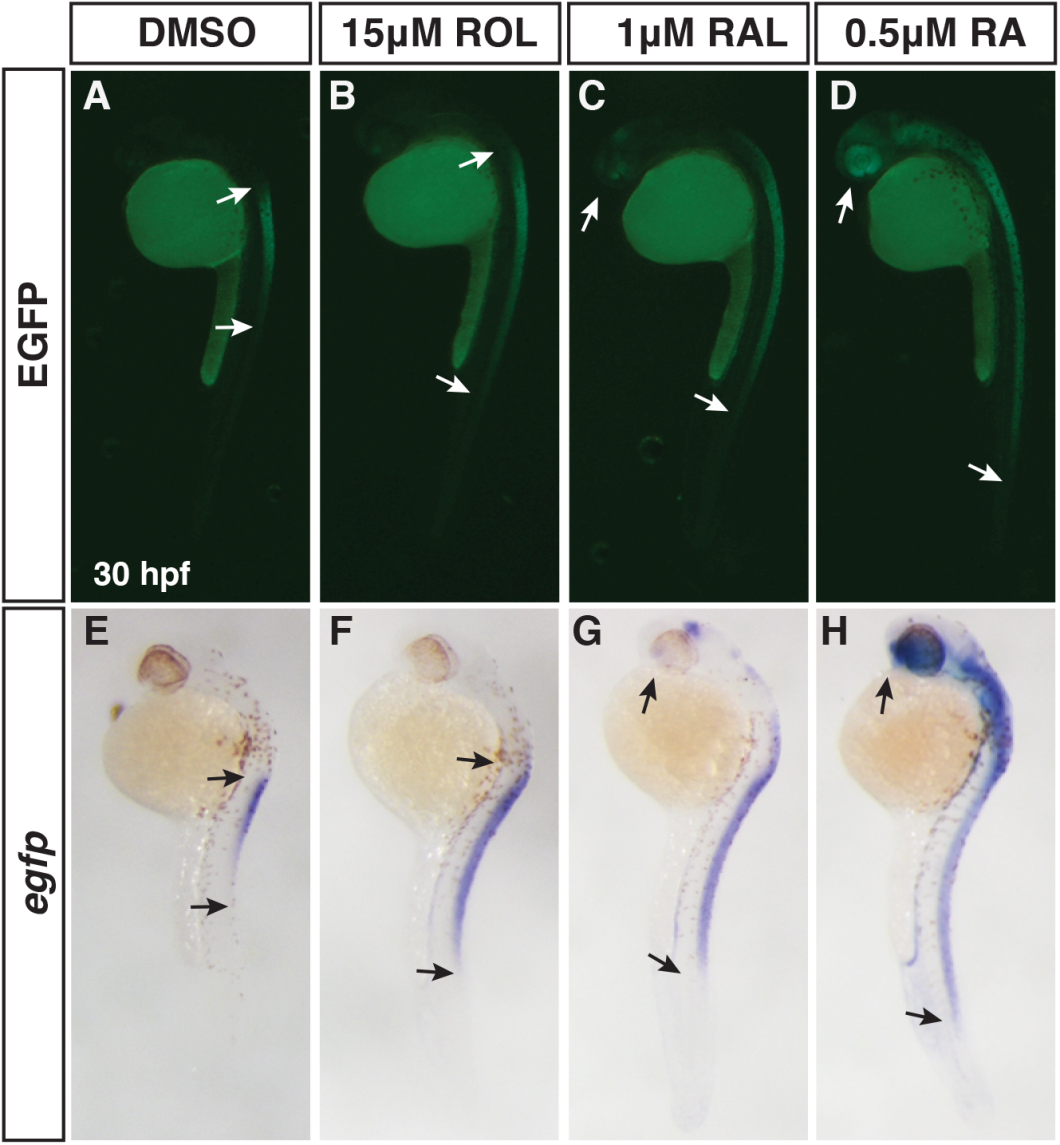
Differential abilities of ROL, RAL and RA to promote RA signaling. (A-D) EGFP fluorescence in *Tg(12XRARE-ef1a*:*EGFP)* ^*sk72*^ embryos after treatment with 15 μM ROL, 1 μM RAL, and 0.5 μM RA beginning at 24 hpf. (E-H) ISH for *egfp* expression in *Tg(12XRARE-ef1a*:*EGFP)* ^*sk72*^ embryos after treatment with 15 μM ROL, 1 μM RAL, and 0.5 μM RA beginning at 24 hpf. Arrows indicate the boundaries of expression. Views are lateral with dorsal right and anterior up.

**Fig 7:**
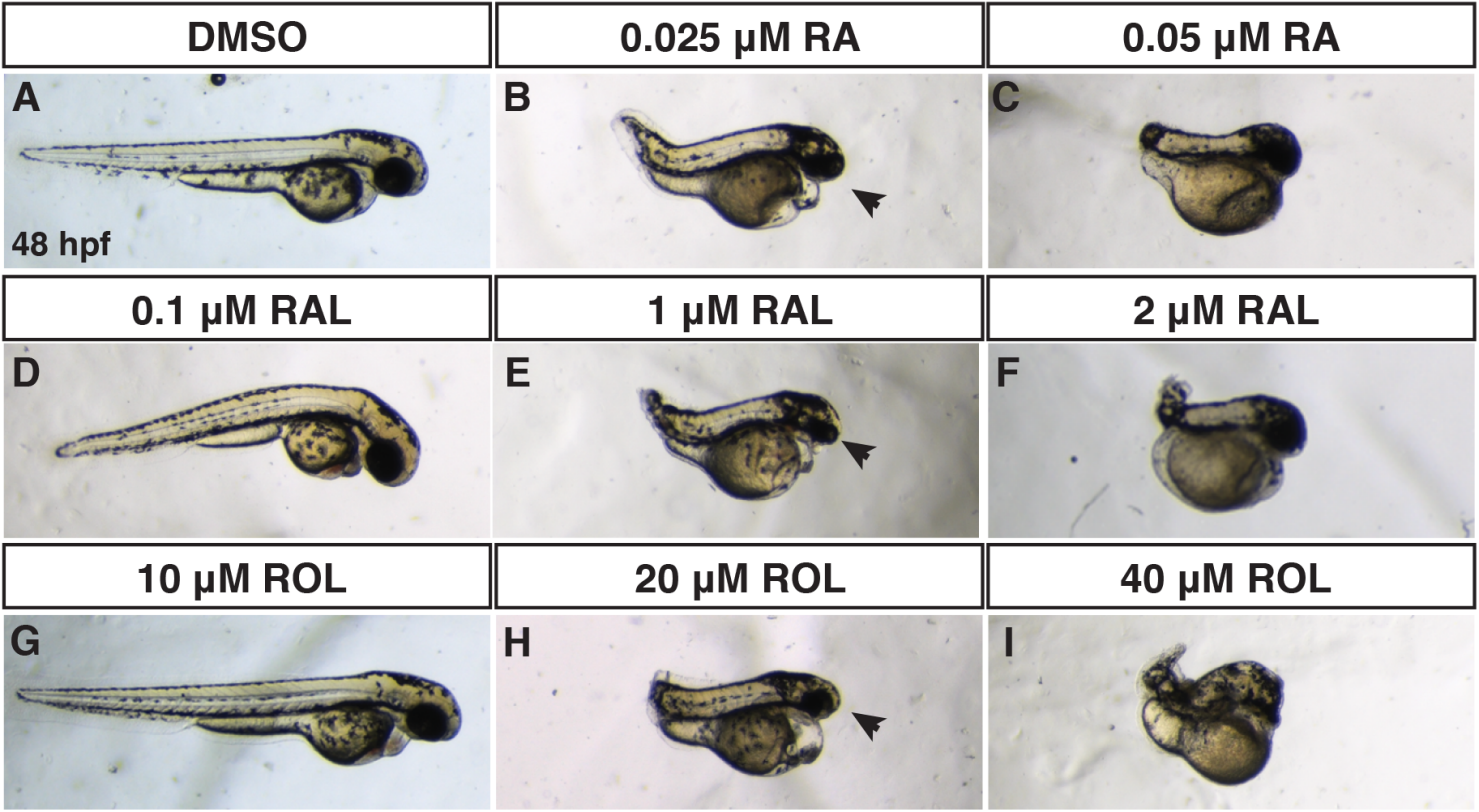
Comparison of RA, RAL, and ROL teratogenicity. (A-J) Embryos at 48 hpf that were treated from sphere stage through 24 hpf with DMSO, ROL, RAL, and RA. (D,G) Low concentrations of RAL (0.1 μM) and ROL (10 μM) did not cause significant overt defects. (B,E,H) Intermediate concentrations of RAL (1 μM) and ROL (20 μM) and the lower concentration of RA (0.025 μM) produced truncated embryos that still had eyes (arrows). (C,F,I) 0.05 μM RA, 2 μM RAL, and 40 μM ROL produced severely truncated embryos without eyes.

**Fig 8:**
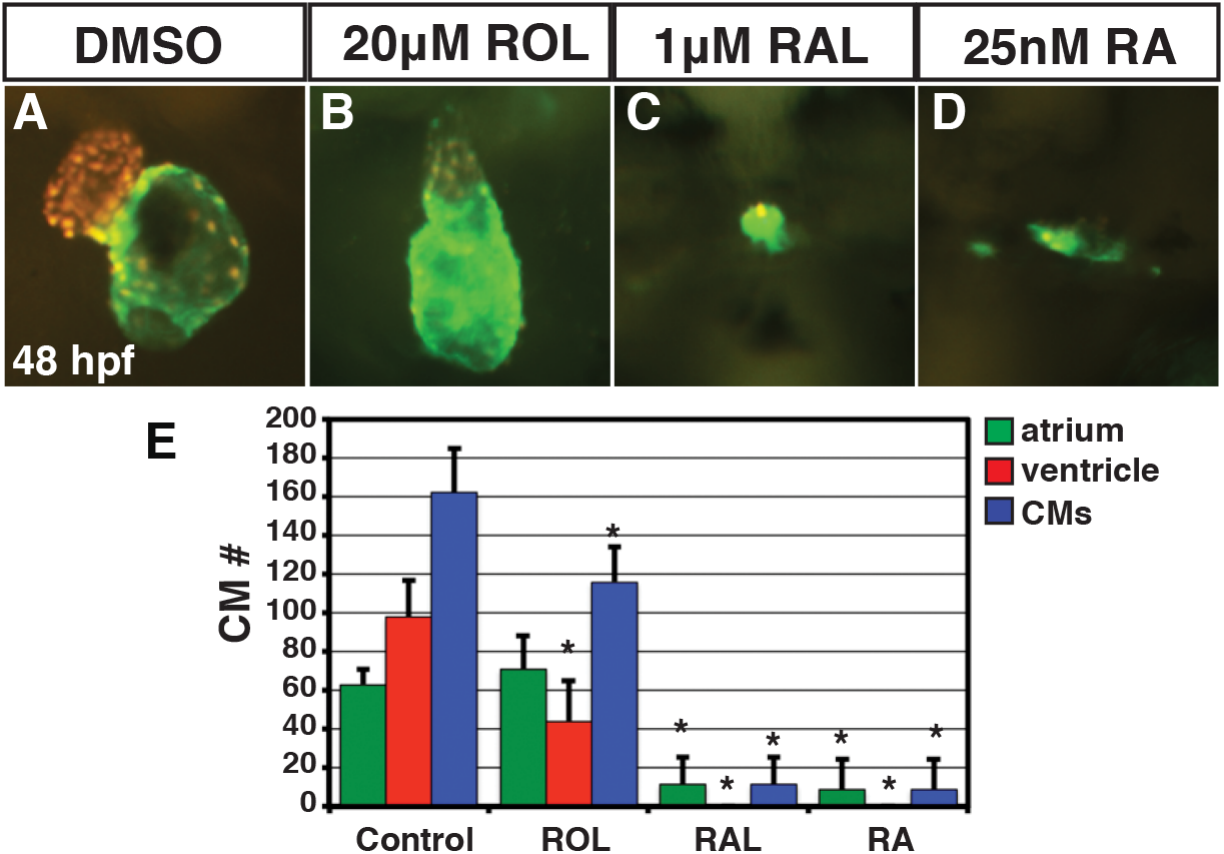
ROL, RAL, and RA eliminate CMs. (A-D) Hearts from *Tg(-5*.*1myl7*:*DsRed-NLS)* ^*f2*^ embryos treated with DMSO, 20 μM ROL, 1 μM of RAL and 0.025 μM RA. Images are frontal views. Red indicates ventricle. Green indicates atrium. (E) Mean CM number at 48 hpf.

We were intrigued by the observation that ROL treatment at a relatively high concentration (15 μM) caused minor overt malformations, even though this concentration is able to induce expression of the transgenic RA signaling reporter (Fig 5C and 5H). Therefore, we wanted to compare the efficacy of RA reporter induction and the teratogenicity of the different retinoids, as this will enhance our ability to understand and model retinoid embryopathies. We began by treating *Tg(12XRARE-ef1a*:*EGFP)* ^*sk72*^ embryos with different concentrations of ROL, RAL, and RA from 24 to 30 hpf. By comparison to 15 μM ROL, significantly lower concentrations of RAL (1 μM) and RA (0.5 μM) were able to induce reporter expression with RA inducing the most ectopic expression of the reporter (Fig 6A–6H). Given the differences we observed with the retinoid treatments beginning at 24 hpf, we sought to better understand the phenotypic consequences of retinoids when treatments began prior to gastrulation, as vertebrate embryos are very sensitive to excess RA signaling during the early embryonic stages [29, 48, 49]. To do so, we treated embryos from the sphere stage (prior to gastrulation) through 24 hpf with a range of concentrations of ROL, RAL, and RA and analyzed the overt phenotypes at 48 hpf. Surprisingly, we found that it took 800-fold ROL and 40-fold RAL to induce phenotypes overtly equivalent to low concentrations of RA treatment [45] (Fig 7A–7I). While embryos were posteriorized with truncated tails at intermediate concentrations, as would expected, these embryos also had significantly reduced heads, eyes, and tails (Fig 7B, 7E and 7H). Higher concentrations were more severely teratogenic and induced a greater truncation of the embryos with complete loss of the eyes (Fig 7C, 7F and 7I). Treatments initiated slightly later at the shield stage gave a similar trend, but the embryos were less affected, supporting the different sensitivity of embryos to excess retinoids at early stages of development (S5 Fig) [49]. Although the highest concentrations used were able to largely eliminate the heart, we noticed that embryos treated with the intermediate concentration of the ROL still had beating hearts, even though in most other aspects they overtly appeared phenotypically similar to intermediate concentrations of RAL and RA treatment. Therefore, we examined the consequences of retinoid treatment on cardiac morphology and CM number at 48 hpf with these intermediate doses using *Tg(-5*.*1myl7*:*DsRed-NLS)* ^*f2*^ embryos [38, 40]. These analyses revealed that persistent treatment with 1μM RAL and 0.025 μM of RA results were largely able to eliminate both atrial and ventricular CMs (Fig 8C and 8D), consistent with what we have demonstrated previously [45]. However, 20 μM ROL produced embryos that had linearized hearts with reduced ventricular number and no difference in atrial number (Fig 8A, 8B and 8E), which is similar to treatments with lower concentrations of RA [31, 45]. Therefore, these results demonstrate that while ROL can produce teratogenic phenotypes consistent with RA embryopathy, ROL is a relatively poor teratogen compared to RAL and RA in zebrafish.

### A conserved teleost enhancer can promote expression in the eye through Pax2 binding sites

**Fig 9:**
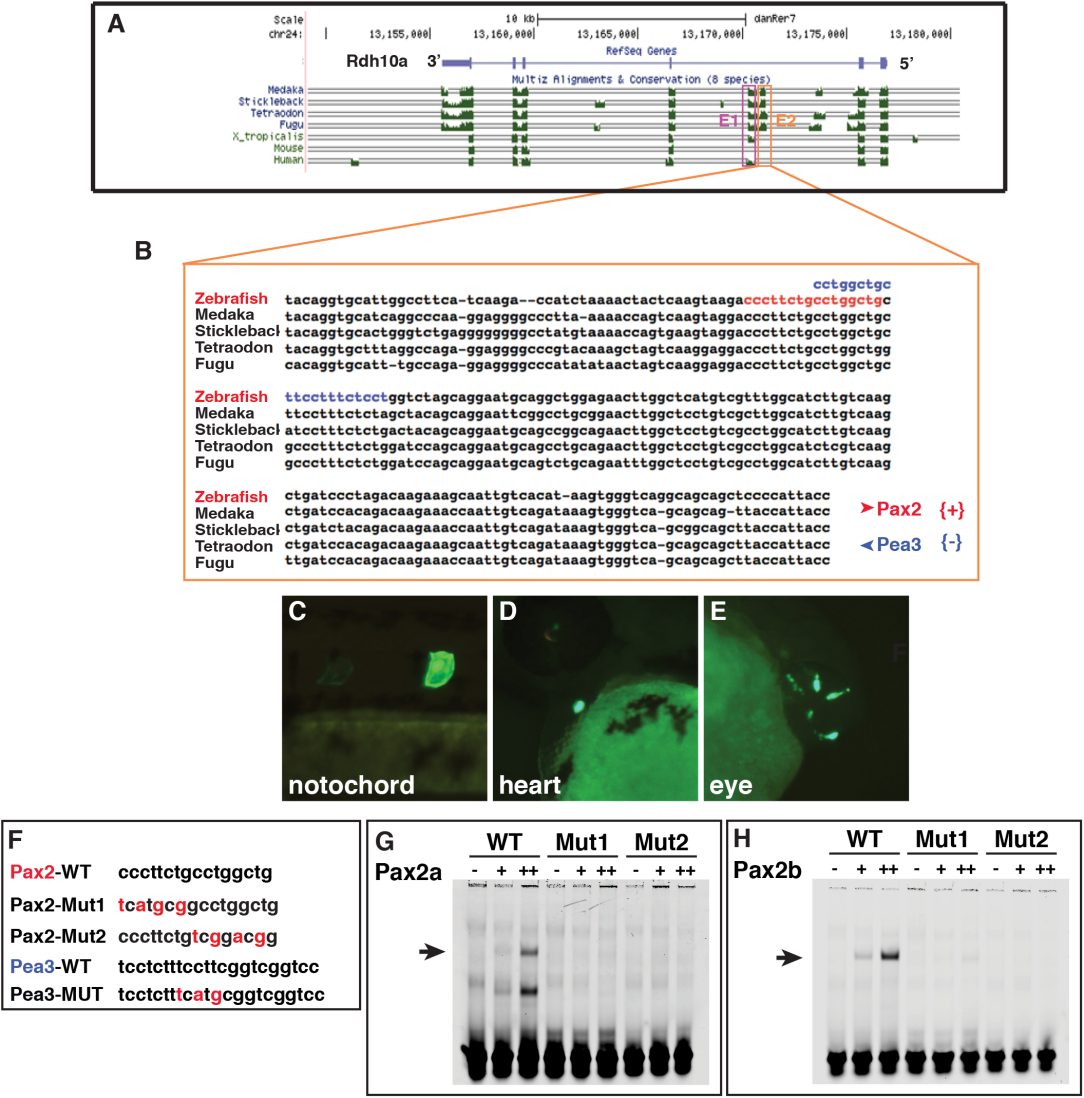
Zebrafish Pax2 proteins bind a putative Rdh10a enhancer. (A) Alignment of vertebrate *rdh10* genes to the zebrafish *rdh10a* taken from the UCSC genome browser. (B) Sequence alignment of the E2 enhancer in teleosts. Red nucleotides indicate the predicted Pax2 binding site. Blue indicates the Pea3 binding site (C-E) Representative images from transient transgenic embryos with *rdh10a* E2-enhancer expression in the notochord, heart, and eye. (F) Mutations that were made in the promoter of the *rdh10a* E2-enhancer and the oligos used for EMSA. (G,H) EMSA with the WT and mutated Pax2 sites and zebrafish Pax2a and Pax2b.

While it has been shown that Rdh10 genes have conserved expression in tissues during development and are negatively regulated by RA signaling in vertebrates [23, 29, 31, 32], the transcriptional mechanisms underlying its regulation are still not understood. To shed light on the Rdh10a regulatory elements responsible for its expression, we carried out *in silico* analysis using the UCSC browser (genome.ucsc.edu). These analyses revealed the presence of two adjacent conserved non-codiing sequences (CNSs) located between exons 2 and 3 (Fig 9A), but no observable conservation was found immediately upstream of the transcriptional start site or downstream of the 3’ UTR. The E1 element is conserved from fish to humans, though absent in mice, and the E2 element is conserved only in the teleosts (Fig 9B). To investigate if these conserved sequences were able to regulate expression of a reporter gene in the same territories where Rdh10a is endogenously expressed, we cloned these elements upstream of a minimal thymidine kinase promoter into a EGFP reporter vector using the Tol2kit [50]. Embryos injected with either of these constructs were monitored for up to 3 days post-fertilization (dpf). Despite the increased conservation of the E1 element, this construct did not show any EGFP expression, suggesting in this context it is not sufficient to promote expression. However, embryos injected with the zebrafish E2 reporter had expression in the notochord, heart and eye at 24 and 48 hpf (Fig 9C–9E and Table 1). Although it is not clear that there is endogenous *rdh10a* expression in the notochord or heart, *rdh10a* is endogenously expressed in the retina of the eye [33] (http://zfin.org). In order to identify possible binding sites recognized by known transcription factors present in the E2 sequence, we used Genomatix (http://www.genomatix.de/cgi-bin/eldorado.main.pl) and Cis-BP (http://cisbp.ccbr.utoronto.ca) [51]. This analysis revealed the presence of multiple putative transcription factor binding sites, with Pax2 binding sites catching our attention because of *pax2* expression in the eye (Fig 9B). To determine if the putative Pax2 binding site is responsible for the eye expression from the *rdh10a* E2 reporter, we mutated the Pax2 binding site and found that this abrogated the expression in the eye from the reporter (Table 1). However, mutation of an adjacent putative Pea3 site, which is also expressed in the eye [52], did not eliminate expression in the eye (Table 1). To determine if Pax2 can bind the putative element, we performed *in vitro* electrophoretic mobility shift assays (EMSAs) with the zebrafish Pax2 paralogs, Pax2a and Pax2b, since both are expressed in the eye during development. Both Pax2a and Pax2b were able to bind the WT-Pax2 site, but were unable to bind the mutated sites (Fig 9F–9H). Since these results suggested that Pax2 genes might be regulating *rdh10a* expression from this enhancer, we investigated if Pax2 genes are required for *rdh10a* expression in the eye. Although a previous study investigating RA signaling in the eye indicated that in *pax2a/no isthmus* (*noi*) mutants *rdh10a* expression was normal [33], the possibility that Pax2b functions redundantly with Pax2a to regulate *rdh10a* expression in the eye was left open. However, we found that co-injection of a *pax2a* MO, which alone produces defects, in particular a loss of midbrain-hindbrain boundary, indistinguishable from *noi* mutants [53, 54], and a previously published *pax2b* MO [55] did not affect *rdh10a* expression in the eye (S6 Fig). Furthermore, using the heat-shock inducible Pax2a line [56], we also did not find a difference in expression when Pax2 expression was increased at the 16 somite stage (S7A and S7B Fig). Therefore, while Pax2 sites are required to promote expression in the eye of a transient transgenic putative *rdh10a* enhancer, our data suggest that *in vivo* Pax2 TFs may not be required to regulate *rdh10a* expression and/or there is compensation from other factors.

**Table 1:** Summary of enhancer analysis.

Construct	n	notochord	heart	eye
WT-*rdh10a*-E2	181	57 (31%)	18 (10%)	9 (5%)
*pax2*-deletion	40	10 (25%)	2 (5%)	0 (0%)
*pax2*-mut1	97	26 (27%)	14 (14%)	0 (0%)
*pax2*-mut2	58	9 (16%)	2 (3%)	0 (0%)
*pea3*-mut	79	24 (30%)	5 (6%)	2 (3%)

## Discussion

Here, we examined the conserved requirements of a zebrafish Rdh10 as an enzyme that contributes to the production of embryonic RA. Using loss of function, we find that Rdh10a is required for RA signaling in early zebrafish embryos, but that the phenotypes are relatively mild compared to perturbation of Aldh1a2. Conversely, gain of function experiments demonstrate that increased Rdh10a expression can enhance the sensitivity to ROL and production of RA. Furthermore, despite the conservation of Rdh10a function, ROL is a relatively mild teratogen in zebrafish compared to RAL or RA. Therefore, our results suggest Rdh10a has a conserved function in the production of RA in early vertebrate embryos, but that Rdh10a has more limited requirements in zebrafish embryos compared to its mammalian homologs.

Comparing our observations to the previously published work in *Xenopus* and mice [23, 26, 28, 29], the phenotypes indicative of mild RA loss of function in Rdh10a deficient zebrafish embryos are more similar to Rdh10 deficient *Xenopus* embryos than the Rdh10 mutant mice. Like what we observed with Rdh10a zebrafish embryos, Rdh10 deficient *Xenopus* embryos have phenotypes that are consistent with moderate loss of RA signaling and are not as anteriorized as with Aldh1a2 deficiency [29]. Rdh10 mutant or knockout mice do have variable background-dependent phenotypes [26, 28]. However, the most severe phenotypes are almost indistinguishable from Aldh1a2 knockout mice [23], suggesting a significant requirement for Rdh10 in mammals. One of the reasons for the difference in requirement for Rdh10 enzymes in zebrafish and *Xenopus* compared to mammals may be that RAL, not ROL, is the major retinoid stored in these lipid filled embryos [43, 44, 47]. Therefore, Rdh10a, or other Rdh enzymes, may not be needed for significant production of RAL in anamniotes compared to amniotes, which have comparably higher amounts of ROL [44, 57]. Alternatively, it is possible that other zebrafish Rdh enzymes contribute more to embryonic RAL production than in mammals and these enzymes are able to compensate for the loss of Rdh10a. For instance, it is possible that Rdh10b contributes to the production of embryonic RA, but we have not yet investigated the level of redundancy. We also cannot rule out that the phenotypes we observe are relatively modest because Rdh10a is not completely depleted from the embryos. However, a failure to completely deplete Rdh10 protein could also explain the modest phenotypes observed in *Xenopus* [29]. Although Rdh10a deficiency produces modest loss of RA signaling alone, it is interesting that Rdh10a depletion is able to enhance the severity of phenotypes by perturbing Aldh1a2 genetically (*nls* mutants) or pharmacologically (DEAB treatment). Therefore, despite the modest phenotypic defects alone, our results are consistent with Rdh10a being part of the proposed core of conserved enzymes that contributes to the production of RAL [23, 29] and is necessary for embryonic RA signaling in vertebrates.

In support of a conserved function of Rdh10a in the production of RA from ROL during early embryogenesis, we found that injection of *rdh10a* mRNA alone was sufficient to promote expression of a RA signaling reporter. Interestingly, there were not strong overt indications of patterning defects at earlier stages. Although we found that concomitant ROL treatment with *rdh10a* mRNA injection enhanced RA signaling similar to what was found in *Xenopus* [29], the posteriorization and tail defects were still not as severe as treatment with low concentrations of RA. We were surprised to find that it was necessary to use 800-fold the concentration of ROL to obtain similar overt phenotypic defects as RA. Moreover, even though our data support that Rdh10a is limiting within the early zebrafish embryo, the supraphysiolocial levels of ROL that it took to induce posteriorization and tail defects suggests that the lack of sufficient Rdh activity is not a major contributing factor to the ineffective ROL teratogenicity. Because hypervitaminosis A can occur in humans and mammalian models [13, 58], we were somewhat surprised by the relatively weak teratogenicity of ROL compared to RAL or RA. However, in human cases of hypervitaminosis A, the individuals were reported to ingest significantly high concentrations of vitamin A [3]. Furthermore, in experiments using mammalian models, animals were given high concentrations of vitamin A over several days [10–12]. Together, these observations suggest that the conversion to RA beginning with ROL may be somewhat inefficient or that other factors are also limiting in zebrafish. In support of the latter idea, the work in *Xenopus* found that *Rdh10* mRNA did not produce overt phenotypes alone, but could anteriorize embryos when co-injected with Aldh1a2 mRNA, indicating these two enzymes together are necessary to enhance the production of RA [29]. Our results suggest that the production of RAL must still be somewhat minimal, as relatively low concentrations of RAL were able to produce RA signaling gain of function similar to RA treatment. One possible explanation for the reduced potency of ROL is that factors like Rbp4 and Stra6, which contribute to the uptake, storage, and release of ROL [59–61], are limiting in the zebrafish embryo. Thus, there may be a reduced ability of ROL to penetrate embryonic tissues and minimal, or slow, release of RAL. Other possibilities are that the decreased potency of ROL treatment is a result of oxidation occurring during the treatment or the induction of negative feedback that impacts the amount of RAL and RA produced [30].

The amount of RA in vertebrate embryos is tightly regulated through conserved feedback regulation on RA synthesizing and degrading enzymes [30]. Increased RA signaling in embryos promotes the expression of requisite negative regulators of RA production, in particular Dhrs3 [31, 36, 62], a SDR that converts RAL back to ROL for eventual storage, and Cyp26a1 [31, 37, 48, 63–65], which degrades RA. Conversely, excess embryonic RA signaling negatively regulates expression of the RA producing Aldh1a2 [27, 31, 41, 63, 65]. One of the key observations leading to the hypothesis that Rdh10 enzymes are part of the core conserved RA production code is that RA signaling negatively regulates its expression during early embryogenesis [23, 29, 31, 32]. Although the responsiveness to RA signaling is conserved in Rdh10 vertebrate homologs, how this negative regulation occurs is still not understood. Conserved elements in the promoter have not been an immediate key to its transcriptional regulation. However, RA signaling is required for proper patterning of the retina [33]. Our results suggest that Pax2 may contribute to the regulation of *rdh10a* expression in the eye, although they do indicate that if this is the case other factors must compensate or be required in parallel *in vivo*.

In conclusion, our study indicates that Rdh10a is part of the conserved core embryonic RA production machinery in zebrafish. Our studies have provided insight into the conserved function of the RA production machinery in vertebrates and the teratogenicity of vitamin A. Ultimately, these findings will be useful tools for comparing the evolution and conserved mechanisms of RA production, signaling, and feedback to better model retinoid teratogenicity in vertebrates.

## Materials and Methods

### Ethics Statement

All zebrafish husbandry and experiments were performed in accordance with protocols approved by the Institutional Animal Care and Use Committee (IACUC) of Cincinnati Children's Hospital Medical Center.

### Zebrafish husbandry and transgenic lines

Zebrafish (*Danio rerio*) were raised and maintained as previously described [66]. The following transgenic lines were used: *Tg(-5*.*1myl7*:*DsRed-NLS)* ^*f2*^ [40], *Tg(12XRARE-ef1a*:*EGFP)* ^*sk72*^ [67], and *Tg(hsp70l*:*pax2a)* ^*x23*^ [56].

### In situ hybridization (ISH)

Whole-mount single and double ISH were carried out using standard methods with NBT/BCIP (Roche) and INT/BCIP (Roche) solutions [68]. Probes for the following genes were used: *hoxb5b* (ZDB-GENE-000823-6), *dhrs3a* (ZDB-GENE-040801-217), *cyp26a1* (ZDB-GENE-990415-44), *eng2a* (ZDB-GENE-980526-167), *egfp* (accession number: JQ064510.1), *egr2b* (formerly *krox20*; ZDB-GENE-980526-283), and *pax2a* (ZDB-GENE-990415-8).

### MO, mRNA, and DNA injections

Translation (*rdh10a* MO1–5’-GATGTTCATCACCATGTTTAATGCC) and splice-blocking (*rdh10a* MO2–5’-TAAAAAGAGGCTCACCCAGAAGTGC) MOs were used to target *rdh10a* (S1 Fig). To knockdown Rdh10a, a cocktail of both MOs was used at 2.5 ng MO1 and 0.7 ng MO2. Sequences for *pax2a* and *pax2b* were reported previously. 9 ng *pax2a* MO and 7.5 ng *pax2b* MO were used for injections. The dose of *pax2a* MO used produced a phenotype indistinguishable from the *noi* mutants [53, 54]. For all injection experiments, 2 ng of *p53* MO was used to help suppress non-specific MO-induced cell death [69]. Full-length *rdh10a* was cloned into pCS2+ and capped mRNA was made using a Sp6 Message Machine Kit (Ambion). 200 and 300 pg of *rdh10a* mRNA were used for experiments, as indicated in the Results. For the rdh10a-E2 reporter, 20 pg of DNA was co-injected with 25 pg Tol2 mRNA.

### Reverse transcriptase real time quantitative PCR (RT-qPCR)

Embryos were harvested and RNA isolated as previously described [31]. RT-qPCR for *myl7*, *amhc*, *vmhc*, *hoxb5b*, *dhrs3a*, *cyp26a1* and *egfp* was performed using standard PCR conditions in a Bio-Rad CFX PCR machine with Power SYBR Green PCR Master Mix (Applied Biosystems). Primer sequences have been reported previously [31]. Expression levels were standardized to *ef1*α expression and all the data were analyzed using the 2^–ΔΔCT^ Livak Method. All experiments were performed in a biological triplicate.

### Length Measurements

Length of *egfp* expression and *egr2b* expression in rhombomere 5 was measured in arbitrary units (A.U.) using ImageJ. Statistical analysis was performed as previously described [38].

### Imaging of zebrafish hearts and cell counting

Immunohistochemistry, cell counting and statistical analysis were done as previously described [38]

### Retinoid and DEAB treatments

ROL (Sigma R7632), RAL (Sigma R2500), RA (Sigma R2625) and DEAB (Sigma D86256) were dissolved in DMSO. For DEAB, embryos were treated from the sphere stage to 24 hpf and subsequently analyzed at 48 hpf. For ROL, RAL, and RA, embryos were treated in the dark at concentrations and length of time as indicated in the Results.

### Heat-shock experiments

Hemizygous *Tg(hsp70l*:*pax2a)* adults were crossed to wild-type adults. The resulting embryos were raised to the 16s stage and heat-shocked at 37°C for 30 min in a Bio-Rad C1000 PCR machined. Embryos were then allowed to develop until 24 hpf, when they were fixed and processed for ISH. To distinguish between non-transgenic and transgenic siblings, ISH for *pax2a* was performed as described above.

### EMSA

EMSA was performed essentially as reported in [70], with the following modifications. Target oligonucleotides were designed with a 15 bp 3’ (ACATTCGCGCAGATC) extension. A common complementary oligonucleotide to the 15 bp extension was synthesized with a 5’ LI-COR IRDye 700 (IDT). The oligonucleotides were annealed and the ends filled with Klenow (New England Biolabs). Proteins for EMSA were made using the TnT SP6 Quick Coupled Transcription/Translation System (Promega). Gels were imaged using an Odyssey CLx LI-COR imager.

### Statistical analysis

To assess whether the means of two groups are statistically different from each other, the Student’s t-test was used. A *p* value of <0.05 was considered statistically significant.

## Supporting Information

S1 Fig Schematic of *rdh10a* MOs used in experiments. *(A)* Schematic of the *Rdh10a* locus. White bar indicates 5’ and 3’UTR. Black bar indicates exons. Green and red bars indicate MOs. *Rdh10a* MO1 targets the translation start site. *Rdh10a* MO2 targets the donor site of the second exon-intron boundary. Purple arrowheads indicate the location of the primers used for the PCR in B. (B) PCR on cDNA from control and *rdh10a* MO2-injected embryos. *Rdh10a* MO2 injection causes some retention of the second intron (red arrow and red X), as well as other improper splice variants. As it was not completely efficient, it was co-injected with the translation blocking MO. (C) Schematic of the effect that improper splicing from *rdh10a* MO2 has on the Rdh10a protein. A premature stop codon (black X) occurs prior to the dehydrogenase domain (blue box).
(TIF)

S2 Fig Specificity controls for the Rdh10a MOs. (A-D) ISH for *egfp* expression in *Tg(12XRARE-ef1a*:*EGFP)* ^*sk72*^ control sibling (n = 13), Rdh10a deficient (n = 21), *rdh10a* mRNA injected (n = 20), and Rdh10a deficient + *rdh10a* mRNA injected embryos (n = 14). Brackets indicate the length of *egfp* expression in the spinal cord. Images are lateral views at 24 hpf. (E) Measurements of *egfp* expression length in the spinal cord of *Tg(12XRARE-ef1a*:*EGFP)* ^*sk72*^ embryos in arbitrary units (AU).
(TIF)

S3 Fig Rdh10a depletion enhances defects caused by inhibition of Aldh1a2. (A) Control sibling embryo treated with DMSO. (B) Embryo treated with 0.05 μM DEAB (a suboptimal concentration) that has mild defects indicated of loss of RA signaling. (C) Rdh10a deficient embryo. (D) Rdh10a deficient embryos treated with the suboptimal concentration of DEAB results in an interaction that produces defects reminiscent of Rdh10a depleted *nls* mutant embryos (Fig 4). The head is enlarged (brackets) and the border between the hindbrain and anterior spinal cord is accentuated (arrow), indicating the hindbrain is anteriorized.
(TIF)

S4 Fig Functional interaction between Rdh10a depletion and Aldh1a2 inhibition in the hindbrain. (A-D) ISH for *eng2a* (blue), which marks the midbrain-hindbrain boundary, and *egr2b* (red), which marks rhombomeres (r) 3 and 5. Control DMSO treated (n = 13), Rdh10a deficient embryos (n = 13), 0.05 μM DEAB treated (n = 12), and Rdh10a deficient + 0.05 μM DEAB embryos (n = 14). (E) Schematic and measurements of r5 length in arbitrary units (AU). Asterisks indicate a statistical significant difference between embryos treated with the DMSO versus all the other conditions. Hashtag indicates a statistically significant difference between embryos injected with *rdh10a* MOs or treated with DEAB versus the embryos treated with DEAB and injected with *rdh10a* MOs.
(TIF)

S5 Fig Comparison of ROL, RAL and RA teratogenicity from treatments beginning at the shield stage. (A-I) Embryos at 48 hpf that were treated from shield stage through 24 hpf with DMSO, ROL, RAL, and RA. (D,G) Low concentrations of RAL (0.1 μM) and ROL (10 μM) did not cause significant overt defects. (B,E,H) Intermediate concentrations of RAL (1 μM) and ROL (20 μM) and the lower concentration of RA (0.025 μM) produced truncated embryos that still had eyes (arrows). (C,F,I) 0.05 μM RA, 2 μM RAL, and 40 μM ROL produced severely truncated embryos with reduced eyes.
(TIF)

S6 Fig *Rdh10a* expression in Pax2 deficient embryos. (A-D) *Rdh10a* expression at 24 hpf of control sibling, Pax2a deficient, Pax2b deficient, and Pax2a+Pax2b deficient embryos. There was no discernible effect on *rdh10a* expression in the eyes (arrows) between control and Pax2 deficient embryos.
(TIF)

S7 Fig *Rdh10a* expression in heat-shocked *Tg(hsp70l*:*pax2a)* embryos. (A, B) *Rdh10a* expression (blue) at 24 hpf in heat-shocked control sibling non-transgenic and hemizygous *Tg(hsp70l*:*pax2a)* embryos. ISH for *pax2a* (red) was performed to distinguish between control sibling and transgene carriers. 12 of the 27 embryos did not have ectopic *pax2a* expression indicating they were non-trangenic, while 15 of the 27 embryos has ectopic *pax2a* expression indicating they were the *Tg(hsp70l*:*pax2a)* embryos. There was no discernible difference between *rdh10a* expression in the eyes (arrows) of control embryos and embryos with increased Pax2a.
(TIF)
