# Supplementary figures and images for: Rdh10a Provides a Conserved Critical Step in the Synthesis of Retinoic Acid during Zebrafish Embryogenesis
Source: PLoS One. 2015 Sep 22;10(9):e0138588. doi: 10.1371/journal.pone.0138588 (PMC4578954; doi:10.1371/journal.pone.0138588)

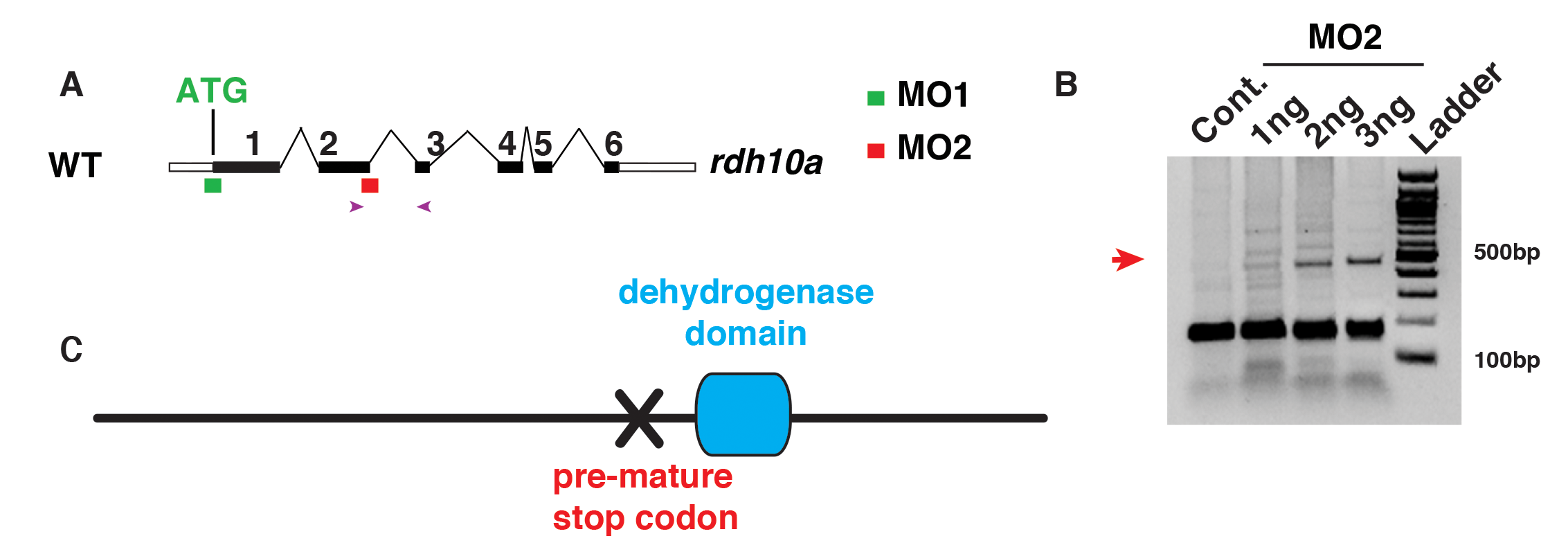

Supplement: S1 Fig — (A) Schematic of the Rdh10a locus. White bar indicates 5’ and 3’UTR. Black bar indicates exons. Green and red bars indicate MOs. Rdh10a MO1 targets the translation start site. Rdh10a MO2 targets the donor site of the second exon-intron boundary. Purple arrowheads indicate the location of the primers used for the PCR in B. (B) PCR on cDNA from control and rdh10a MO2-injected embryos. Rdh10a MO2 injection causes some retention of the second intron (red arrow and red X), as well as other improper splice variants. As it was not completely efficient, it was co-injected with the translation blocking MO. (C) Schematic of the effect that improper splicing from rdh10a MO2 has on the Rdh10a protein. A premature stop codon (black X) occurs prior to the dehydrogenase domain (blue box). (TIF) [file pone.0138588.s001.tif]

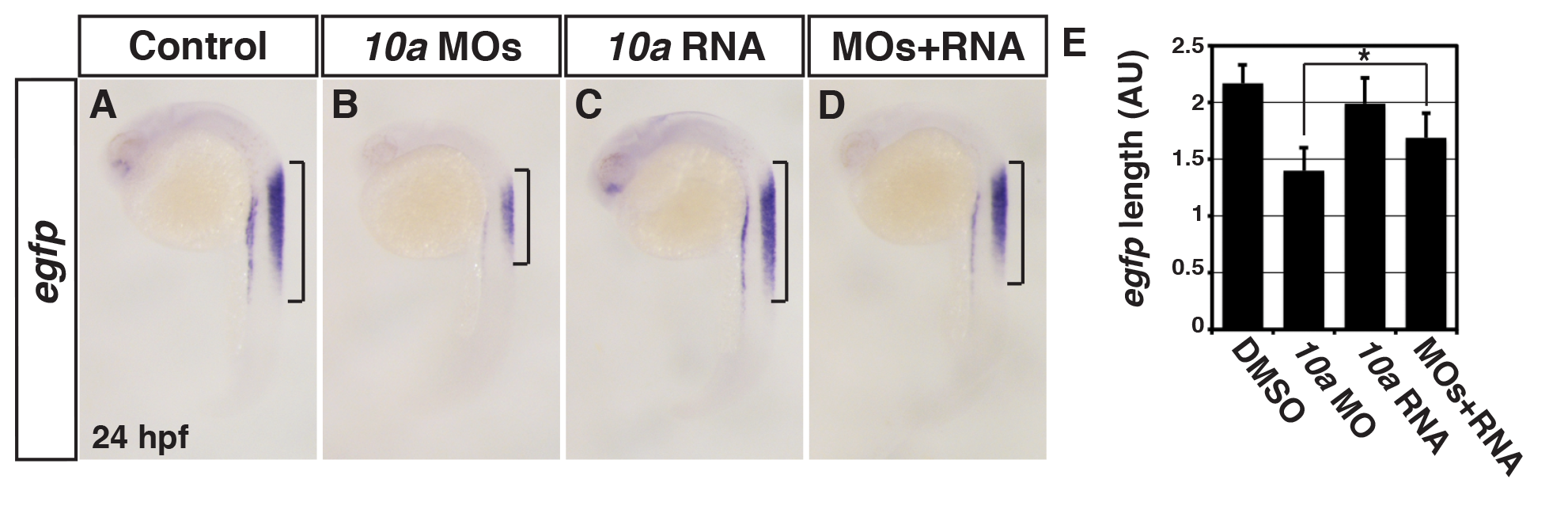

Supplement: S2 Fig — (A-D) ISH for egfp expression in Tg(12XRARE-ef1a:EGFP) sk72 control sibling (n = 13), Rdh10a deficient (n = 21), rdh10a mRNA injected (n = 20), and Rdh10a deficient + rdh10a mRNA injected embryos (n = 14). Brackets indicate the length of egfp expression in the spinal cord. Images are lateral views at 24 hpf. (E) Measurements of egfp expression length in the spinal cord of Tg(12XRARE-ef1a:EGFP) sk72 embryos in arbitrary units (AU). (TIF) [file pone.0138588.s002.tif]

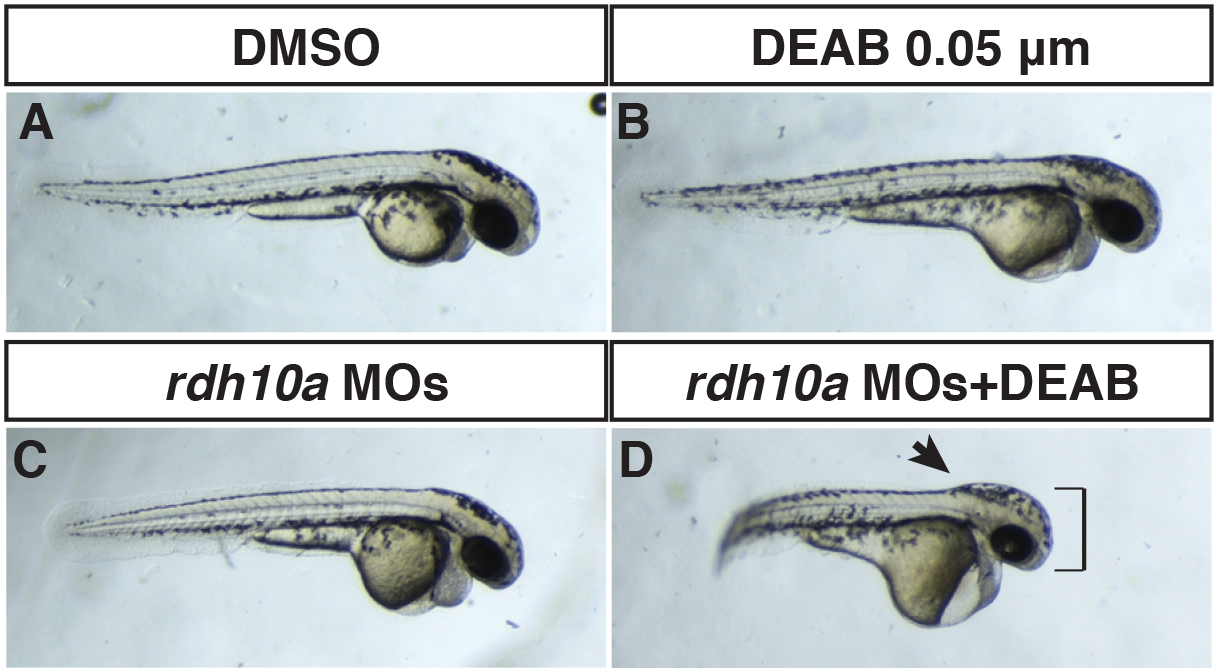

Supplement: S3 Fig — (A) Control sibling embryo treated with DMSO. (B) Embryo treated with 0.05 μM DEAB (a suboptimal concentration) that has mild defects indicated of loss of RA signaling. (C) Rdh10a deficient embryo. (D) Rdh10a deficient embryos treated with the suboptimal concentration of DEAB results in an interaction that produces defects reminiscent of Rdh10a depleted nls mutant embryos (Fig 4). The head is enlarged (brackets) and the border between the hindbrain and anterior spinal cord is accentuated (arrow), indicating the hindbrain is anteriorized. (TIF) [file pone.0138588.s003.tif]

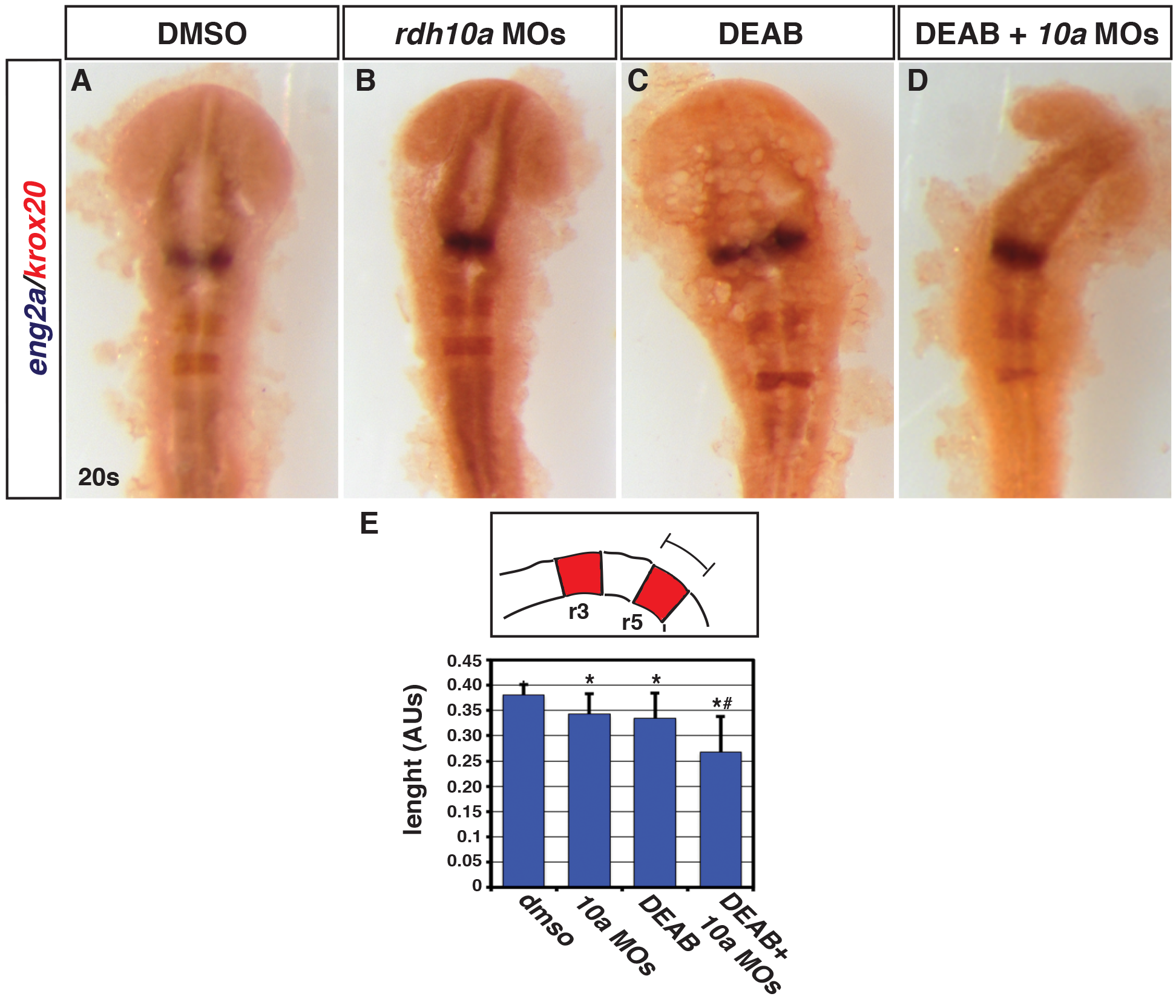

Supplement: S4 Fig — (A-D) ISH for eng2a (blue), which marks the midbrain-hindbrain boundary, and egr2b (red), which marks rhombomeres (r) 3 and 5. Control DMSO treated (n = 13), Rdh10a deficient embryos (n = 13), 0.05 μM DEAB treated (n = 12), and Rdh10a deficient + 0.05 μM DEAB embryos (n = 14). (E) Schematic and measurements of r5 length in arbitrary units (AU). Asterisks indicate a statistical significant difference between embryos treated with the DMSO versus all the other conditions. Hashtag indicates a statistically significant difference between embryos injected with rdh10a MOs or treated with DEAB versus the embryos treated with DEAB and injected with rdh10a MOs. (TIF) [file pone.0138588.s004.tif]

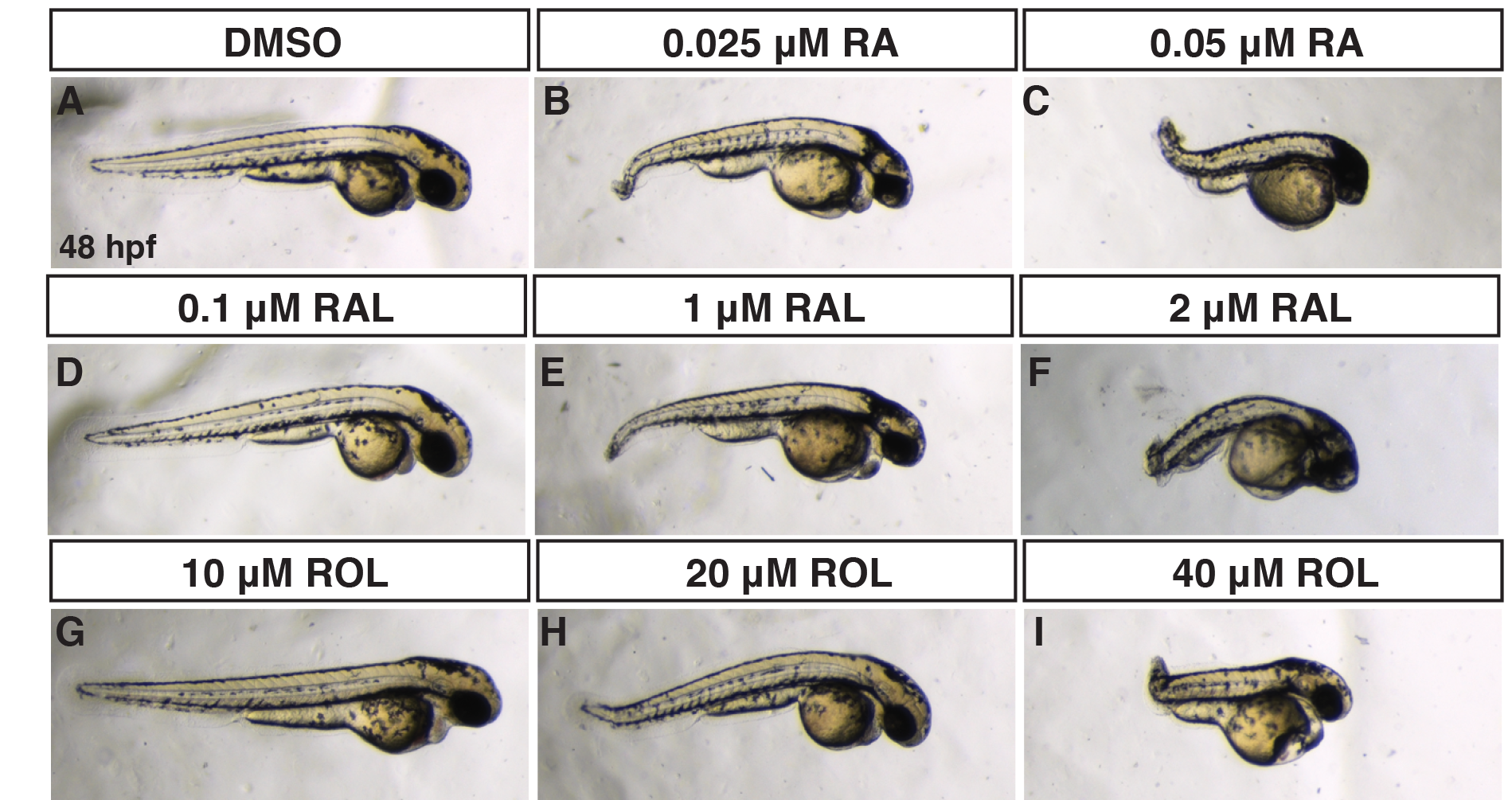

Supplement: S5 Fig — (A-I) Embryos at 48 hpf that were treated from shield stage through 24 hpf with DMSO, ROL, RAL, and RA. (D,G) Low concentrations of RAL (0.1 μM) and ROL (10 μM) did not cause significant overt defects. (B,E,H) Intermediate concentrations of RAL (1 μM) and ROL (20 μM) and the lower concentration of RA (0.025 μM) produced truncated embryos that still had eyes (arrows). (C,F,I) 0.05 μM RA, 2 μM RAL, and 40 μM ROL produced severely truncated embryos with reduced eyes. (TIF) [file pone.0138588.s005.tif]

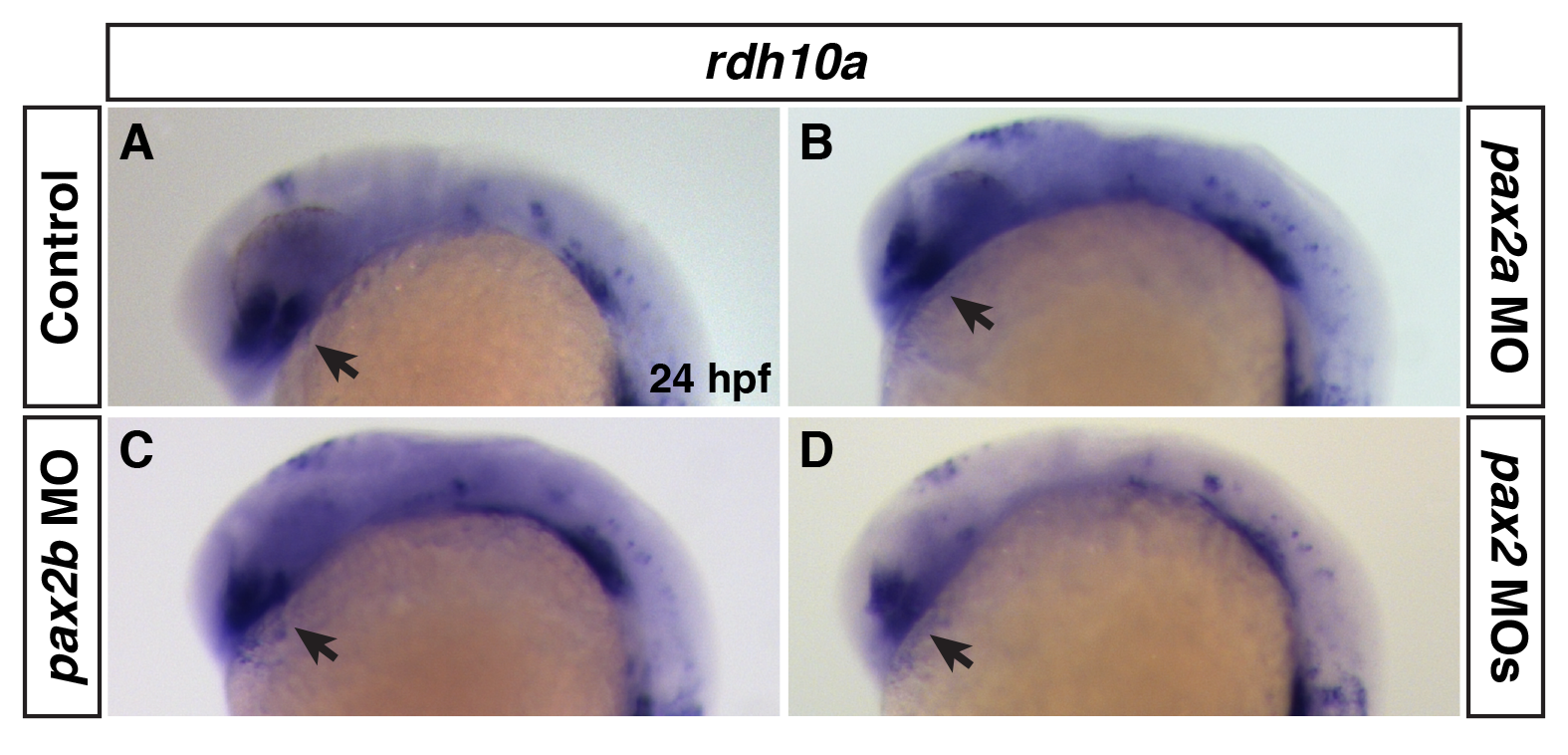

Supplement: S6 Fig — (A-D) Rdh10a expression at 24 hpf of control sibling, Pax2a deficient, Pax2b deficient, and Pax2a+Pax2b deficient embryos. There was no discernible effect on rdh10a expression in the eyes (arrows) between control and Pax2 deficient embryos. (TIF) [file pone.0138588.s006.tif]

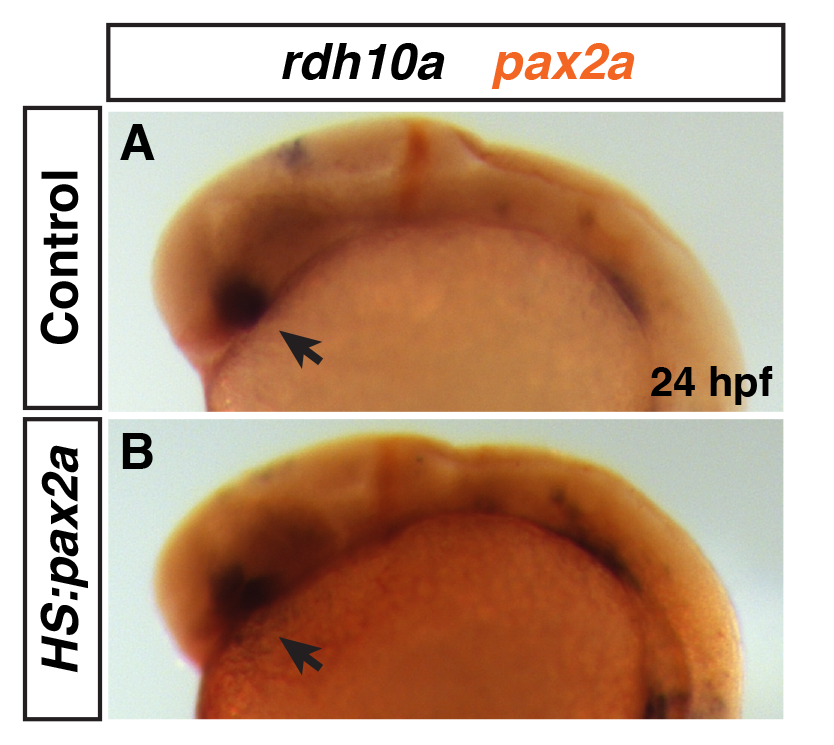

Supplement: S7 Fig — (A, B) Rdh10a expression (blue) at 24 hpf in heat-shocked control sibling non-transgenic and hemizygous Tg(hsp70l:pax2a) embryos. ISH for pax2a (red) was performed to distinguish between control sibling and transgene carriers. 12 of the 27 embryos did not have ectopic pax2a expression indicating they were non-trangenic, while 15 of the 27 embryos has ectopic pax2a expression indicating they were the Tg(hsp70l:pax2a) embryos. There was no discernible difference between rdh10a expression in the eyes (arrows) of control embryos and embryos with increased Pax2a. (TIF) [file pone.0138588.s007.tif]
